# FABP4 promotes invasion and metastasis of colon cancer by regulating fatty acid transport

**DOI:** 10.1186/s12935-020-01582-4

**Published:** 2020-10-19

**Authors:** Wenying Tian, Wenjia Zhang, Yan Zhang, Tianyue Zhu, Yuting Hua, Hui Li, Qinglin Zhang, Min Xia

**Affiliations:** 1 Department of Gastroenterology, Wuxi People’s Hospital Affiliated to Nanjing Medical University, 299 Qing Yang Road, Wuxi, 214023 Jiangsu People’s Republic of China; 2 Department of Gastroenterology, The Second Affiliated Hospital of Nantong University, Nantong, 226001 Jiangsu China

**Keywords:** Colon cancer, Fatty acid binding protein 4, Metastasis, Lipid, Epithelial–mesenchymal transition, AKT

## Abstract

**Background:**

The prognosis of colon cancer is poor for metastasis, while the mechanism, especially adipocytes related, is not yet clear. The purpose of this study is to determine the effects of fatty acid binding protein 4 (FABP4), a transporter for lipids, on colon cancer progression.

**Methods:**

The distribution of lipids and FABP4 was tested in the colon cancer tissues and adjacent normal tissues, and their relationship was also verified in vitro. Experiments about cellular invasion, migration and proliferation were performed to detect the impacts of FABP4 on the biological behaviors of colon cancer, and the positive results were checked in vivo. Meanwhile, the regulatory role of FABP4 in the energy and lipid metabolism was evaluated by the levels of triglyceride, ATP, LDH, glycerol and NEFA. At last, GO and KEGG analysis based on FABP4 overexpressed cells was performed, and the AKT pathway and epithelial–mesenchymal transition (EMT)-related proteins were determined by Western blot.

**Results:**

Higher accumulation of lipids and stronger FABP4 transcription were observed in colon cancer tissues. Having been incubated with adipose tissue extract and overexpressed FABP4, colon cancer cells demonstrated enhanced lipid accumulation. In functional experiments, co-culture with adipose tissue extract significantly enhanced the invasion and migration of colon cancer cells, as well as the energy and lipid metabolism, and all these processes were reversed by FABP4 inhibitor. In addition, the metastasis of FABP4-overexpressed colon cancer cells was also significantly enhanced in vitro and in vivo. In terms of mechanism, the bioinformatics analysis showed that FABP4 was enriched in 11 pathways related to metabolic processes in FABP4 overexpressed cells. Finally, FABP4 overexpression improved EMT progression of colon cancer, as evidenced by the upregulation of Snail, MMP-2 and MMP-9, the downregulation of E-cadherin. The expression of p-Akt was also elevated.

**Conclusion:**

FABP4 overexpression could increase FAs transport to enhance energy and lipid metabolism, and activate AKT pathway and EMT to promote the migration and invasion of colon cancer cells.

## Background

Colon cancer is a common malignant tumor worldwide. About 1,200,000 new cases and more than 600,000 related deaths are reported annually [1]. Recent studies have shown the critical role of tumor microenvironment in the invasion and metastasis of cancers [2]. The tumor microenvironment is a complex system of hypoxia, low pH and high pressure, composed of tumor cells, stromal cells (including fibroblasts, inflammatory cells, fat cells, glial cells and etc.) and extracellular matrix. It regulates a variety of biological behaviors of tumor cells, including proliferation and metastasis [2, 3]. When cultured in vitro, tumor cells will present different biological profiles, even functional deficits and tissue specificity [4].

Recent studies have shown that intraperitoneal tumor can preferentially metastasize to adipose tissues, but the underlying mechanism is still unclear [3, 5]. Adipose tissues are composed of abundant mature adipocytes that can secrete tumor-related adipokines and participate in the progression of tumors, such as ovarian cancer [3, 5]. Transmission electron microscopy showed that lipid droplets accumulated in colon cancer cells co-cultured with adipocytes, suggesting that adipocytes are the important component in tumor microenvironment [3, 5–7]. It is reported that adipocytes promote homing of ovarian cancer cells to the omentum in nude mice, and the growth of ovarian cancer cells accelerates when co-cultured with adipocytes in vivo and in vitro [3]. It is believed that free fatty acids (FAs), adipokines and signaling factors, which released from adipocytes, play important nutritional and functional supporting roles to impacted in the biological behaviors of various tumors, such as matrix remodeling and epithelial–mesenchymal transition (EMT) [3, 8, 9].

Fatty acid binding proteins (FABPs) are a group of homologous small molecule cytoplasmic proteins that can bind to and transport hydrophobic fatty acids. FABP4, a member of the family, is highly expressed in adipose tissue and macrophages [10]. FABP4 can reversibly bind to, metabolize and transport long-chain fatty acids in metabolic syndrome, inflammation, atherosclerosis and other diseases [3, 11, 12]. The expression of FABP4 was up-regulated in metastatic cancer cells [3]. FABP4 has been found to be expressed at the junction between primary ovarian cancer cells and adipocytes [13]. In prostate and breast cancer, the mRNA and protein levels of FABP4 expression were also significantly up-regulated [14–16].

Mounting evidence has shown the close relationship between obesity and colon cancer [17], but that between adipocytes and colon cancer progression remains unknown, not to mention the related mechanisms. The present study proved, for the first time, the effects of adipocyte-derived FABP4 on the metastasis of colon cancer cells and the related mechanisms, which may provide a new potential therapeutic target against colon cancer progression.

## Methods

### Adipose tissues and preparation of extract

Human adipose tissues were collected from the omentum majus adipose of nontumorous patients immediately after surgery at the Department of Gastrointestinal Surgery, Wuxi People's Hospital of Nanjing Medical University. The adipose tissues were washed with cold PBS, cut into particles with a diameter less than 2 mm under aseptic condition, and then centrifuged to remove red blood cells and debris. Adipose tissues were then incubated in DMEM culture medium at the ratio of 80 mg adipose/mL DMEM medium for 24 h at 37 °C under 5% CO_2_. The extract was obtained by filtering the mixture with 0.45 μM filter filtration (EMD Millipore, Billerica, MA, USA). After incubation with the extract for 24 h, the cancer cells were harvested for subsequent experiments.

### Human colon cancer and adjacent normal tissues, histopathological analysis and immunohistochemistry

Colon cancer tissues were obtained from the patients ready to undergo colon surgery but without prior chemotherapy or radiotherapy in Wuxi People's Hospital from December 2016 to February 2017. Only adenocarcinoma tissues were taken. The adjacent normal tissues were defined by 5 cm above the margin of the cancer, and examined as normal colonic mucosa by pathologists. The fresh tissues were frozen and cut into 8 μm frozen sections. For Oil Red O staining, the frozen sections were air-dried at room temperature, incubated in fresh Oil Red O (Abcam, Cambridge, MA, USA) for 10 min, and rinsed in water. The images were captured under an Olympus optical microscope (Tokyo, Japan).

### Cell culture, drug treatment and adenovirus infection

The human colon cancer cell lines HCT-8 and HCT-116 were cultured in DMEM medium containing 10% fetal bovine serum (FBS) and 1% penicillin and streptomycin (Gibco, Grand Island, NY, USA) at 37 °C under 5% CO_2_ atmosphere. BMS309403 (Sigma-Aldrich, St. Louis, MO, USA) was dissolved in DMSO (Sigma-Aldrich, St. Louis, MO, USA) at 20 mM to prepare a stock solution. In the subsequent studies, 20 μM was used as working solution. The cells were cultured with complete medium in the control group, with medium containing adipose extract (80 mg/mL) in the co-culture group, and with medium containing adipose extract (80 mg/mL) plus 20 μM/L BMS309403 in the co-culture + inhibitor group. Full-length cDNA of an adenovirus-carrying human FABP4 was constructed for expressing FABP4 (ViGene, Shandong, China). The colon cancer cells were infected by the adenovirus in the complete medium (1:100) for 12 h at 37 °C, followed by incubation in fresh complete medium for additional 24 h to 48 h. The infection efficiency was detected by quantitative real-time PCR (qRT-PCR). The cells infected with control adenovirus and FABP4 recombinant adenovirus were assigned to control group and FABP4 overexpression group, respectively.

### Edu assay

A total of 5 × 10^3^ cells were seeded into a 96-well plate and treated for 36 h after an overnight standing (the cells were adhered). Then, 50 μM of Edu medium was added to incubate for 2 h for cell marking. The Edu medium was replaced with cell fixative for cell immobilization. Having been incubated for 2 h at room temperature, the cell fixative was replaced with glycine and incubate for 5 min. Glycine was replaced with Apollo staining solution, followed by an incubation of 30 min in dark at room temperature. The cells were washed with penetrating fluid thrice, with methyl alcohol twice and with PBS once to finish Apollo staining. Then, DNA was stained with Hoechst 33342 reaction solution. The cells stained with fluorescence were observed using a fluorescence microscope (20×, Olympus, Tokyo, Japan) equipped with a digital camera (Canon, Tokyo, Japan). The proliferation of cell was analyzed by Image J software (National Institutes of Health, Bethesda, MD, USA) according to the manufacturer’s instructions (Edu Kit, RiboBio, Guangzhou, China).

### Wound healing migration assay

The colon cancer cells (5 × 10^5^/mL) in exponential growth were isolated and seeded into 6-well tissue culture plates. When a confluent monolayer was formed, the monolayer was scratched with the tips of 200 μL sterile pipettes. Then, the medium was replaced with fresh medium supplemented with 2% FBS. At 0 and 24 h, a microscope (20×, Olympus, Tokyo, Japan) equipped with a digital camera (Canon, Tokyo, Japan) was placed at three random positions to photograph the status of cell migration; and the images were analyzed by Image J. The wound width was used to represent cell migration as means ± sd, and the values were compared with those at 0 h.

### Transwell invasion assay

The invasion model was established by using 24-well Matrigel Invasion Chambers (pore size, 8 μm; Corning, Tewksbury, MA, USA). A total of 5 × 10^4^ colon cancer cells were added as a single cell suspension into 500 μL of serum-free medium containing 0.2% bovine serum albumin (BSA), and seeded onto the upper chambers. The lower chamber was filled with complete culture medium or supplemented with adipose tissue extract. After 24 h, the cells on the surfaces of the upper chambers were scraped off. Then the invading cells were washed with PBS thrice, fixed with formalin for 2 min, and stained with crystal violet staining solution for 30 min at room temperature. The invading cells were photographed under a microscope. Five randomly visual fields were selected from each group and analyzed by Image J.

### Triglyceride assay

A total of 50 mg of tissues were triturated in the mortar containing 1 mL of lysate. The supernatant was collected, heated for 10 min at 70 °C and centrifuged at 2000 rpm for 5 min at room temperature. Triglyceride in the supernatant was calculated using colorimetric assay according to the manufacturer’s instructions (Triglyceride Kit, Applygen, Beijing, China).

### Free FA assay

A total of 8 × 10^4^ cells were seeded into a 96-well plate and treated for 48 h after an overnight standing (the cells were adhered). The medium was collected and centrifuged at 12,000×*g* for 5 min at 4 °C prior to determination. Nonesterified FA (NEFA) in the supernatant was analyzed using colorimetric assay according to the manufacturer’s instructions (Labassay NEFA Kit, Wako, Osaka, Japan).

### ATP assay

A total of 2.5 × 10^5^ cells were seeded into a 6-well plate and treated for 48 h after an overnight standing (the cells were adhered). The cells were harvested and lysed in 100 μL of lysis buffer for 30 min on ice. The supernatant was collected and centrifuged at 12,000×*g* for 5 min at 4 °C prior to determination. ATP in the supernatant was calculated using chemiluminescent assay according to the manufacturer’s instructions (ATP Assay Kit, Beyotime Biotechnology, Shanghai, China).

### LDH assay

A total of 8 × 10^3^ cells were seeded into a 96-well plate and treated for 48 h after an overnight standing (the cells were adhered). The supernatant was collected and centrifuged at 500×*g* for 5 min at room temperature. LDH in the supernatant was calculated using colorimetric assay according to the manufacturer’s instructions (LDH Assay Kit, Beyotime Biotechnology, Shanghai, China).

### Glycerol assay

A total of 5 × 10^3^ cells were seeded into a 96-well plate and treated for 48 h after an overnight standing (the cells were adhered). The medium was collected and centrifuged at 12,000×*g* for 5 min at 4 °C prior to determination. Glycerol in the supernatant was calculated using colorimetric assay according to the manufacturer’s instructions (Glycerol Kit, Applygen, Beijing, China).

### Immunofluorescence staining

The colon cancer cells (1 × 10^5^/mL) in exponential growth were isolated and seeded onto 35 mm glass bottom culture dishes. After treatment, the cells were washed with PBS, fixed with 4% paraformaldehyde and stained with 20 μg/mL Bodipy 493/503 for 30 min in dark at 37 °C, and washed with PBS thrice. The nuclei were stained with Hoechst 33342 (0.5 μg/mL) for 10 min in dark at 37 °C, and observed under a confocal scanning laser microscope (Olympus, Tokyo, Japan). The relative fluorescence intensity was analyzed by Image J. FABP4 fused with EGFP recombinant protein was expressed and purified by Bioworld Biotech (Nanjing, China). The purity of FABP4-EGFP was over 85%. Colon cancer cells were seeded in 6-well plates for 24 h, and then incubated with fresh medium containing FABP4-EGFP for 2 h at 37 °C in a 5% CO_2_ atmosphere. The cells were then washed with PBS thrice. The cells were counterstained using DAPI to visualize the cell nucleus under an immunofluorescence microscope (Olympus, Tokyo, Japan).

### Bioinformatics analysis

The colon cancer cells infected with control adenovirus and FABP4 recombinant adenovirus were subjected to control group and FABP4 overexpression group, respectively. Six samples (three from control group and three from FABP4 overexpression group) were sequenced. The total RNA was extracted according to the manufacturer's instructions and digested with RNAse‐Free DNAse. PCR amplification was then performed using Taq DNA polymeras. RNA samples, with qualified quality, were sequenced on the Illumina Hi-Seq 2500 sequencer (Illumina, SanDiego, CA). Expression levels of each gene across different samples were standardized in order to show its correlation with others. The genes with significant difference in expression were then screened out using |log2FC| > 1 and P < 0.05. Gene ontology (GO) analysis was applied to describe the biological processes and molecular functions involving these genes. Kyoto encyclopedia of genes and genomes (KEGG) pathway analysis was used to find out the enriched pathways.

### Western blot analysis

The protein expression levels of MMP-2, MMP-9, E-cadherin, Snail, FABP4 and p-AKT in colon cancer cells were detected by Western blot. According to the instructions of the BCA kit (Beyotime Biotechnology, Shanghai, China), the total protein of cells was collected. Rat-anti MMP-2 (Cell Signaling Technology, Danvers, MA, USA, 1:1000), MMP-9 (Cell Signaling Technology, Danvers, MA, USA, 1:1000), E-cadherin (Cell Signaling Technology, Danvers, MA, USA, 1:1000), Snail (Cell Signaling Technology, Danvers, MA, USA, 1:1000), FABP4 (Cell Signaling Technology, Danvers, MA, USA, 1:1000), p-AKT (Cell Signaling Technology, Danvers, MA, USA, 1:2000) and the corresponding secondary antibodies were applied. Gay values were analyzed by Image J.

### qRT-PCR assay

Total RNA of the tissues was extracted using TRIzol (Invitrogen, Grand Island, NY, USA) according to the standard TRIZOL method. First-strand cDNA was synthesized from 0.5 μg of RNA per sample by using PrimeScript RT reagent Kit with gDNA Eraser (Takara Biotechnology, Japan). Real-time PCR was performed on an ABI 7500 RT-PCR by using SYBR Premix Ex TaqTM II (Takara Biotechnology, Japan). Table 1 shows the primers for FABP4 and the internal control GAPDH. Gene expression was calculated using the 2^−△△CT^ method.

**Table 1:** Primers for qRT-PCR of FABP4 and GAPDH

Gene	Forward primer sequence (5′–3′)	Reverse primer sequence (5′–3′)
GAPDH	GCACCGTCAAGGCTGAGAAC	TGGTGAAGACGCCAGTGGA
FABP4	TGGGCCAGGAATTTGACGA	CATTTCTGCACATGTACCAGGACAC

### In vivo colon cancer metastasis model in nude mice

Five-week-old female outbred nude mice were bought from Changzhou CAVENS Laboratory Animal Co., Ltd. (Changzhou, China) and raised in a pathogen-free facility with constant temperature and humidity. Colon cancer metastasis mouse model was constructed by injecting 2 × 10^6^/100 μL colon cancer cells through the tail vein. The mice were randomly divided into two groups, in which the colon cancer cells were infected with control adenovirus and FABP4 adenovirus for 24 h, respectively. For imaging assay, all cells were infected with luciferase lentivirus labeled with m-cherry for 48 h. After injection, the distribution of colon cancer cells in organs was analyzed using an in vivo imaging system (Kodak, Effingham, IL, USA). The study was approved by the Ethics Committee of Wuxi People's Hospital of Nanjing Medical University.

### Statistical analysis

All experiments were repeated for three times or more and data were expressed as mean ± sd. The data were analyzed by GraphPad Prism 6.01. Analysis of variance (ANOVA) test was used to compare data from GO analysis and KEGG pathway analysis. Fisher's exact test was used for GO analysis and KEGG pathway analysis. P < 0.05 indicated significant difference.

## Results

### Lipid accumulation in colon cancer cells was related to FABP4-mediated FA transport through the transmembrane

**Fig. 1:**
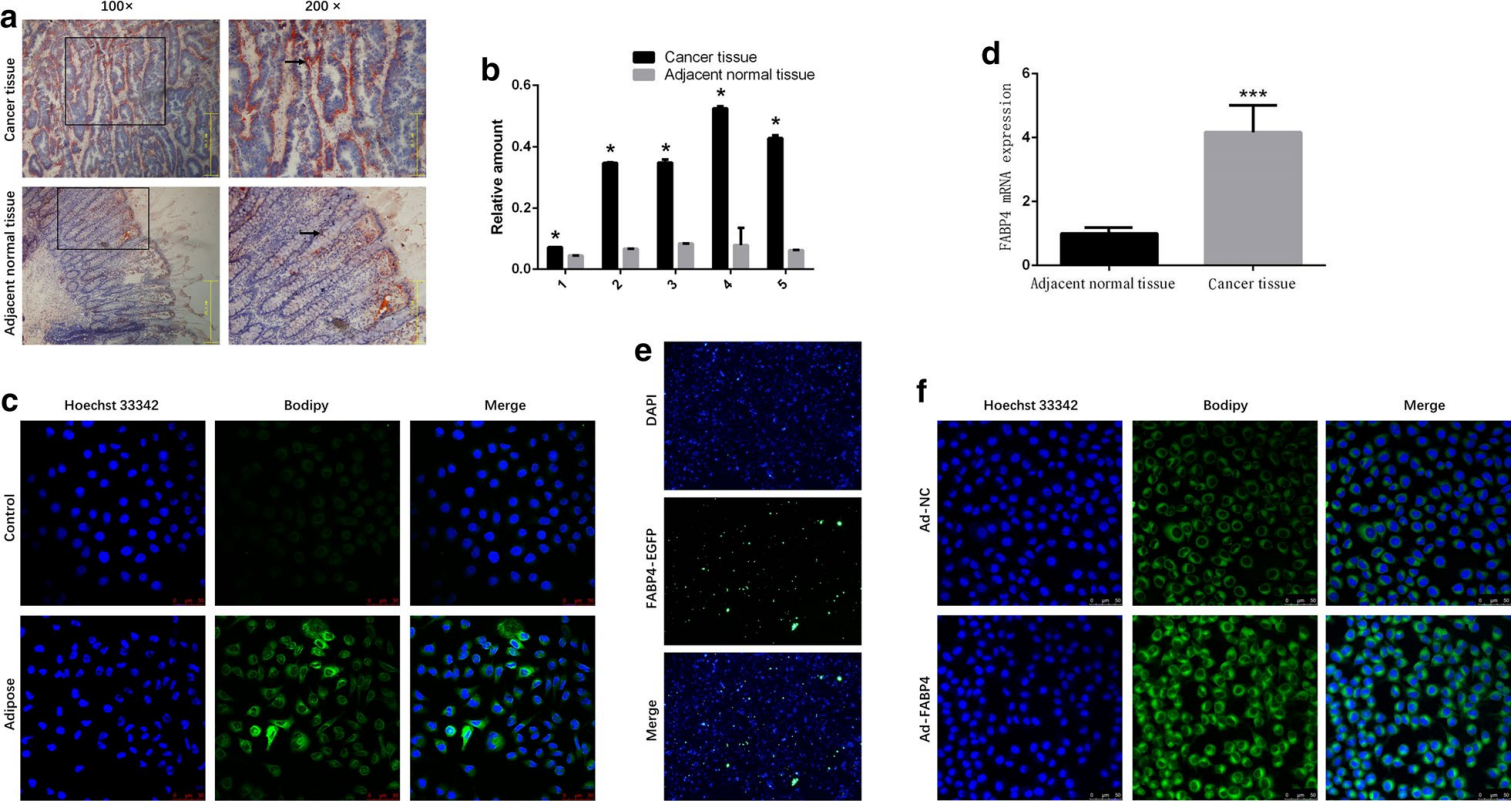
Lipid accumulation in colon cancer cells was related to FABP4-mediated FA transport. **a** The tissues of colon cancer patients (n = 5) were freshly made into 8 μm frozen sections, stained with oil red O solution. The distribution of lipids in human colon cancer tissues and adjacent normal tissues was observed under the microscopy. **b** The supernatant of the colon cancer tissues and adjacent normal tissues lysate were collected for triglyceride colorimetric assays (n = 5). **c** HCT-116 cells were incubated with or without extract of adipose tissues. Lipid distribution was visualized by confocal microscopy analysis, the intracellular lipids were stained by fluorescence dye Bodipy (20 μg/mL), and the nucleus of HCT-116 cells were stained by Hochest33342 (0.5 μg/mL). Magnification: ×400. **d** The mRNA level of FABP4 in colon cancer tissues and adjacent normal tissues were determined by qRT-PCR. **e** Exponential monolayer HCT-116 cells were incubated with synthesized EGFP-tagged FABP4 for 2 h, using EGFP as a control. The intracellular distribution of FABP4 was visualized using the living cell imaging system. Magnification: ×100. **f** In control and FABP4-overexpressed HCT-116 cells, the intracellular lipids were stained by fluorescence dye Bodipy (20 μg/mL) and the nucleus of HCT-116 cells were stained by Hochest33342 (0.5 μg/mL). Magnification: ×400. All the samples were prepared in triplicate, and all experiments were repeated for at least three times. *P < 0.05

To explore the distribution of lipids in colon cancer tissues, we stained the frozen tissue sections with oil red O solution. A large amount of lipids stained bright red accumulated around the colon cancer tissues, but few around the adjacent normal tissues (Fig. 1a). The optical density of lipids in colon cancer tissues was 6.10 ± 0.74 times of that in adjacent normal tissues (Additional file 1: Figure S1A, *P* < 0.05). The triglyceride concentration in colon cancer tissues was significantly higher than in adjacent normal tissues (Fig. 1b, *P* < 0.05).

To investigate the relationship between adipose tissues and colon cancer, we incubated the human colon cancer cell lines HCT-116 with extracts of adipose tissues obtained from the human omentum majus. Lipid accumulation in HCT-116 cells was significantly enhanced (Fig. 1c), and the average optical density of lipids was 2.78 ± 0.27 times of that in control group (Additional file 1: Figure S1B, *P* < 0.05). In our study, we found the transcription level of FABP4 mRNA in colon cancer tissues was 3.17 ± 0.38 times of that in adjacent normal tissues (Fig. 1d, *P* < 0.05). To further explore the relationship between FABP4 and colon cancer, we observed that the synthesized EGFP-tagged FABP4 protein could be taken up by colon cancer cells (Fig. 1e). At the same time, we verified the overexpression of FABP4 at mRNA and protein levels in colon cancer cells (Additional file 1: Figure S2) and found that lipid accumulation was more obvious in FABP4-overexpressed HCT-116 cells compared with control group (Fig. 1f). The average optical density of lipids in FABP4-overexpressed group was 2.00 ± 0.22 times of that in control group (Additional file 1: Figure S1C, *P* < 0.05). The results above indicated that lipid accumulation in colon cancer cells was related to FABP4-mediated FA transport through the transmembrane, a process that promotes lipogenesis.

### FABP4 enhanced the invasion and migration but did not change the proliferation of colon cancer cells

**Fig. 2:**
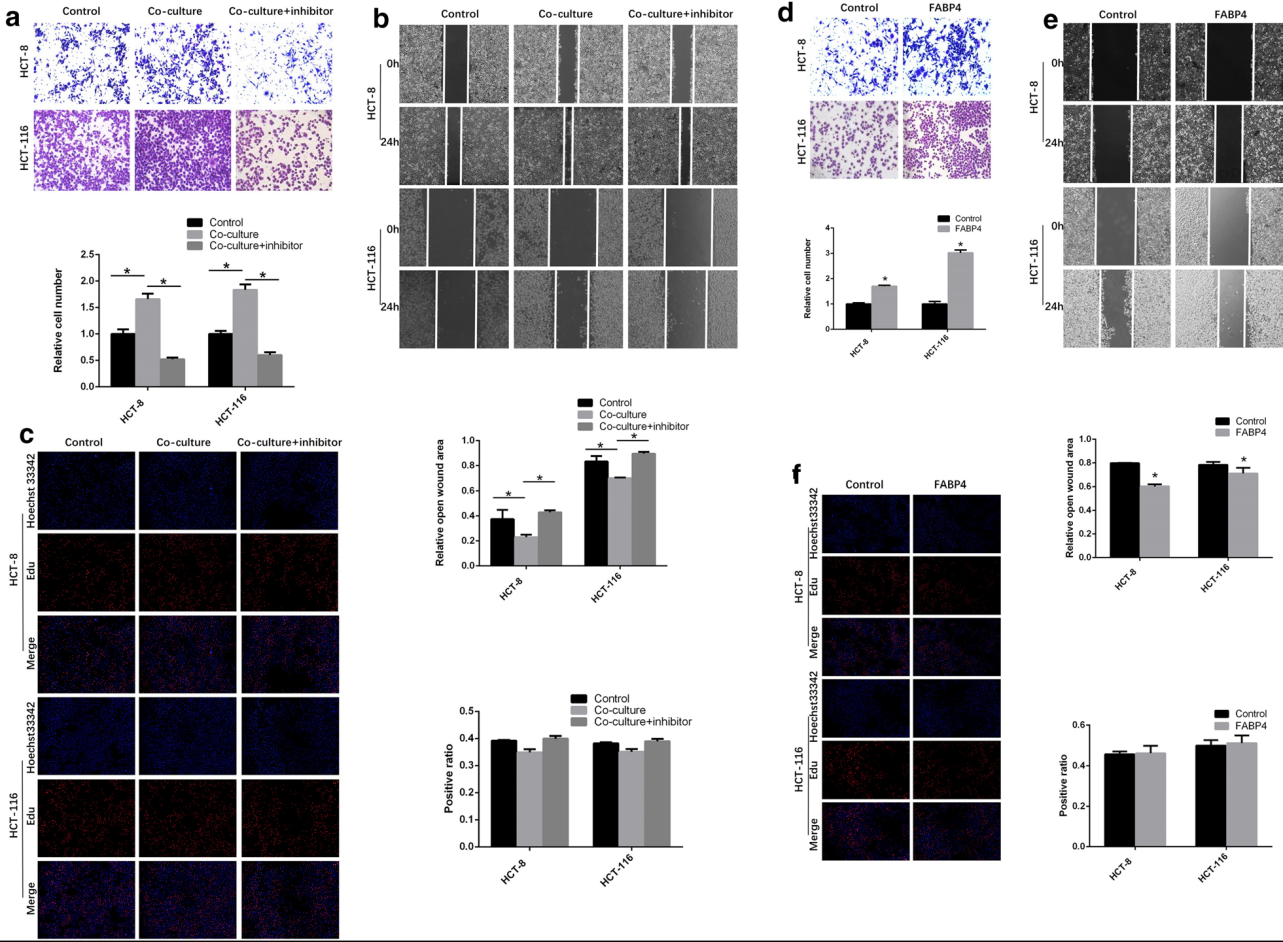
FABP4 enhanced the invasion and migration, but did not change the proliferation of colon cancer cells. **a** HCT-8 and HCT-116 cells were incubated with extract of adipose tissues with or without specific FABP4 inhibitor BMS309403. The invasion of colon cancer cells was determined by Transwell assays. **b** The migration of colon cancer cells was determined by Wound healing assays. **c** The proliferation of colon cancer cells was determined by Edu assays. **d** HCT-8 and HCT-116 cells were infected with FABP4 recombinant adenovirus (1:100) and control adenovirus. The invasion (**d**), migration (**e**) and proliferation (**f**) of the cells were determined by Transwell assays, wound healing assays and Edu assays, respectively. All the samples were prepared in triplicate, and all experiments were repeated for at least three times. Magnification: ×200. *P < 0.05

To investigate the effects of adipose tissues and FABP4 on colon cancer cells invasion and migration, we incubated the human colon cancer cell lines HCT-8 and HCT-116 with extract of adipose tissues with or without specific FABP4 inhibitor BMS309403. In HCT-8 cells, the number of transmembrane colon cancer cells in co-culture group was 1.66 ± 0.08 times of that in control group (Fig. 2a, *P* < 0.05), which means adipose extract enhanced the invasion of colon cancer cells. In co-culture + inhibitor group, the number of transmembrane cells was 31.45% of that in co-culture group (Fig. 2a, *P* < 0.05), which means FABP4 inhibitor suppressed the increased invasion of colon cancer cells. The wound healing assay was conducted to measure migration of colon cancer cells. In HCT-8 cells, the “wound” width at 24 h after scratching in co-culture group was 61% of that in control group (Fig. 2b, *P* < 0.05), which means adipose extract enhanced the migration of colon cancer cells. In co-culture + inhibitor group, the “wound” width was 1.86 ± 0.02 times of that in co-culture group (Fig. 2b, *P* < 0.05), which means FABP4 inhibitor suppressed the increased migration of colon cancer cells. There was no significant difference in proliferation between co-culture group and control group (Fig. 2c, *P* > 0.05), and between co-culture group and co-culture + inhibitor group (Fig. 2c, *P* > 0.05). Similar results were obtained for HCT-116 cells.

To further explore the mechanism of FABP4 in colon cancer metastasis, FABP4 recombinant adenovirus was used to demonstrate the involvement of FABP4 in colon cancer progression. In HCT-8 cells, the number of transmembrane cells in FABP4 overexpression group was 1.70 ± 0.03 times as large as that in control group (Fig. 2d, *P* < 0.05). The “wound” width at 24 h after scratching in FABP4 overexpression group was 76% of that in control group (Fig. 2e, *P* < 0.05). But the proliferation of cancer cells did no change (Fig. 2f, *P* > 0.05). Similar results were observed in HCT-116 cells. Overall, these results demonstrated that FABP4 enhanced the invasion and migration, but did not change the proliferation of colon cancer cells.

### FABP4 enhanced colon cancer metastasis in nude mice

**Fig. 3:**
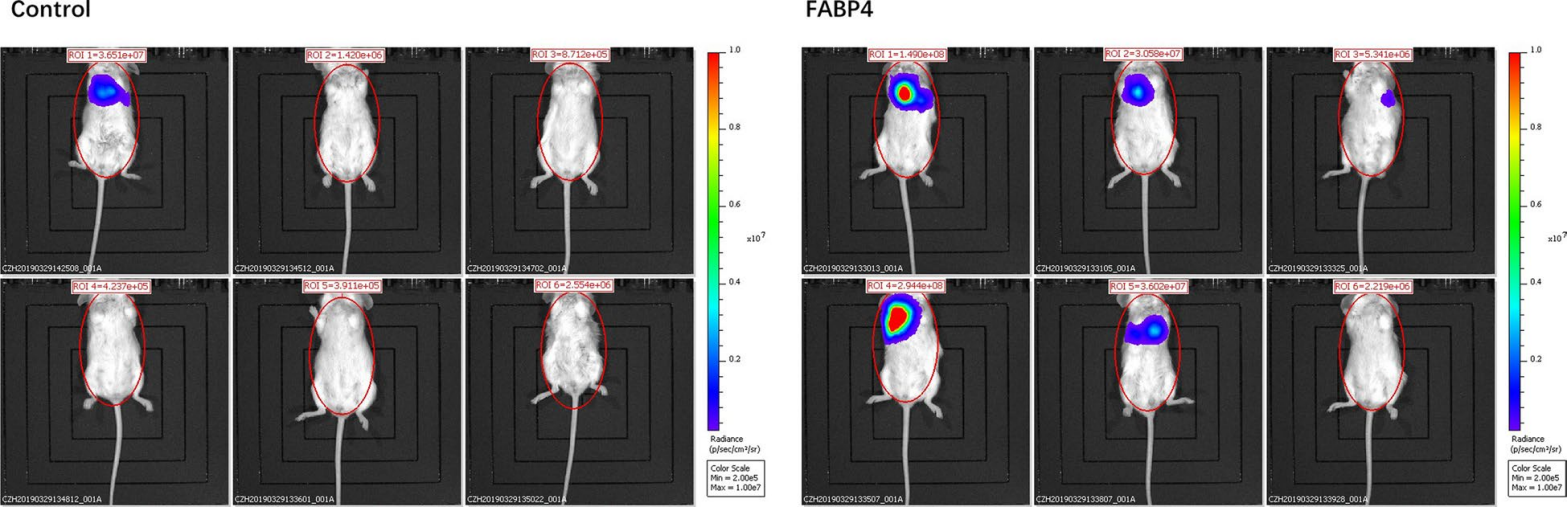
FABP4 enhanced colon cancer metastasis in nude mice. At 30 days after HCT-116 cells was injected into the mice via the tail vein, the distribution of HCT-116 cells was visualized by imaging system. n = 6 per group

The influence of FABP4 on colon cancer metastasis was further confirmed in nude mouse model. At 30 days after HCT-116 cells was injected into the mice via the tail vein, the distribution and accumulation of colon cancer cells were visualized by imaging system (Fig. 3). At day 30, lung metastasis was observed in 83.3% (5/6) of the mice injected with FABP4 overexpressed HCT-116 cells, and 16.7% (1/6) of the mice in control group (*P* < 0.05). No obvious metastasis was observed in other places. A significantly larger number of pulmonary metastases were detected in the mice injected with FABP4-overexpressed HCT-116 cells (FABP4 9 vs. Control 1). The results in vivo were consistent with the in vitro data, showing that FABP4 promoted colon cancer metastasis.

### Adipose extract enhanced energy and lipid metabolism in colon cancer cells

**Fig. 4:**
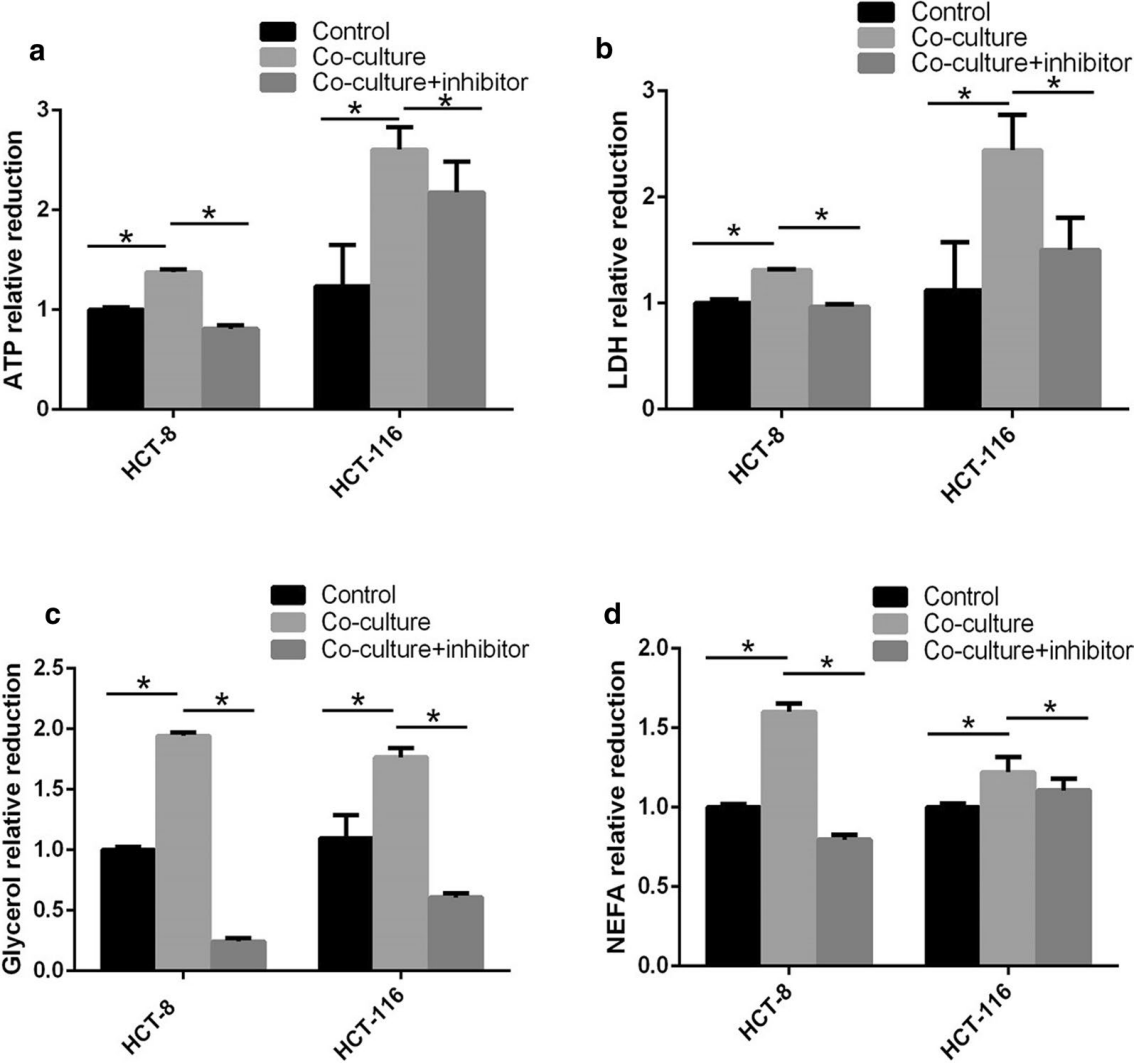
Co-culture with adipose extract enhanced energy and lipid metabolism in colon cancer cells. The HCT-8 and HCT-116 cells were cultured in medium with different treatments (complete medium, medium containing adipose extract (80 mg/mL) and medium containing adipose extract (80 mg/mL) plus 20 μM/L BMS309403). **a** The lysates of cells were collected for intracellular ATP detection. **b**–**d** The culture medium of cells was collected for extracellular LDH, glycerol and NEFA detection. All the samples were prepared in triplicate, and all experiments were repeated for at least three times. *P < 0.05

Energy is the prerequisite of tumor growth and invasion. Based on the above results, we speculated that adipose-induced colon cancer metastasis was closely associated with FAs released by adipocytes, a source of energy metabolism. To investigate whether adipose tissues and FABP4 affect cellular energy metabolism and lipid metabolism, we examined intracellular ATP in colon cancer cells and extracellular LDH, glycerol and non-esterified fatty acid (NEFA) in culture medium supernatant after co-culture with adipose extract containing BMS309403 or not. In HCT-8 cells, the reduction of intracellular ATP in co-culture group was 1.38 ± 0.02 times of that in control group (Fig. 4a, *P* < 0.05); the reductions of extracellular LDH, glycerol and NEFA in co-culture group were 1.31 ± 0.02, 1.94 ± 0.02, 1.60 ± 0.02 times of those in control group, respectively (Fig. 4b–d, *P* < 0.05), indicating adipose extract enhanced energy metabolism and lipid metabolism of colon cancer cells. In co-culture + inhibitor group, the consumption of intracellular ATP in HCT-8 cells reduced by 41%, compared with co-culture group (Fig. 4a, *P* < 0.05); the consumption of extracellular LDH, glycerol and NEFA reduced by 26%, 87%, and 50%, compared with co-culture group, respectively (Fig. 4b–d, *P* < 0.05). Therefore, the significantly enhanced metabolism induced by adipocytes was inhibited by FABP4 inhibitor, suggesting that FABP4 promoted tumor metabolism. Similar results were seen in HCT-116 cells. The results above indicated that adipose extract enhanced energy metabolism and lipid metabolism of colon cancer cells, but this effect was reversed by FABP4 inhibitor, suggesting that FABP4 promoted tumor metabolism.

### FABP4 was enriched in the functions and pathways related to colon cancer

**Fig. 5:**
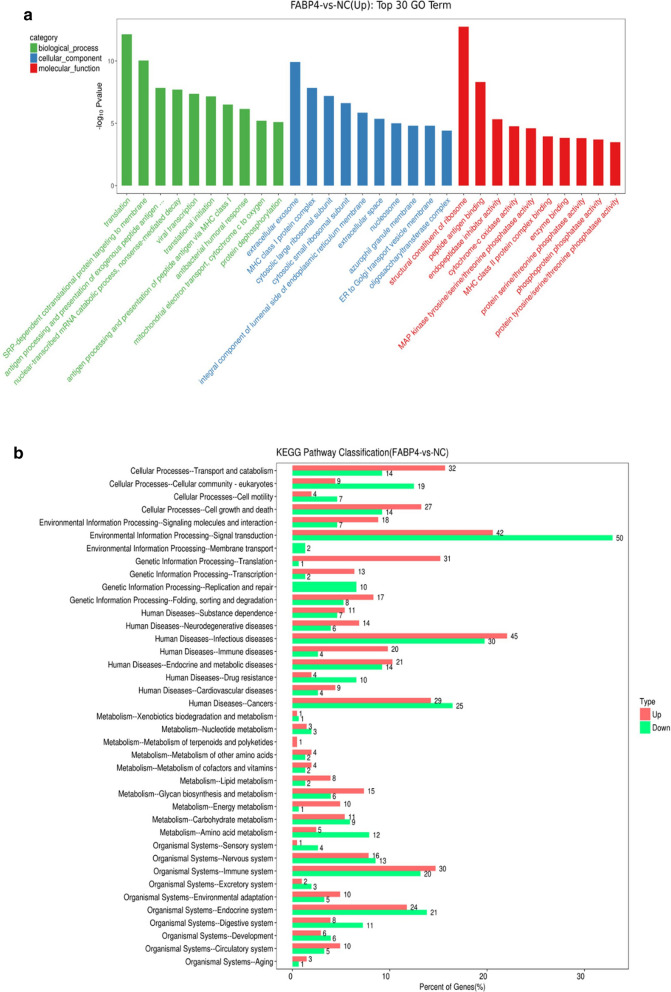
FABP4 was enriched in the functions and pathways related to colon cancer. **a** FABP4-GO network was generated according to the relationships between significant functions and FABP4. The vertical axis is the GOs category and the horizontal axis is the P value of each GO. The lower P value, the closer involvement of FABP4 in GOs related to colon cancer. **b** FABP4-KEGG network was generated according to the relationship between significant signal pathways and FABP4. The vertical axis is the pathway category and the horizontal axis is the P value of each pathway. The lower P value, the more KEGG pathways involving FABP4

The differentially expressed genes in FABP4-overexpressed colon cancer cells were analyzed. The top 30 enriched GO terms (*P* < 0.05) are listed in Fig. 5a. The KEGG analysis suggested that FABP4 was significantly enriched in 39 pathways (Fig. 5b, *P* < 0.05). Among these KEGGs, 11 KEGGs were related to metabolic processes, such as lipid metabolism and energy metabolism.

### FABP4 enhanced EMT in colon cancer cells through AKT pathway

**Fig.6:**
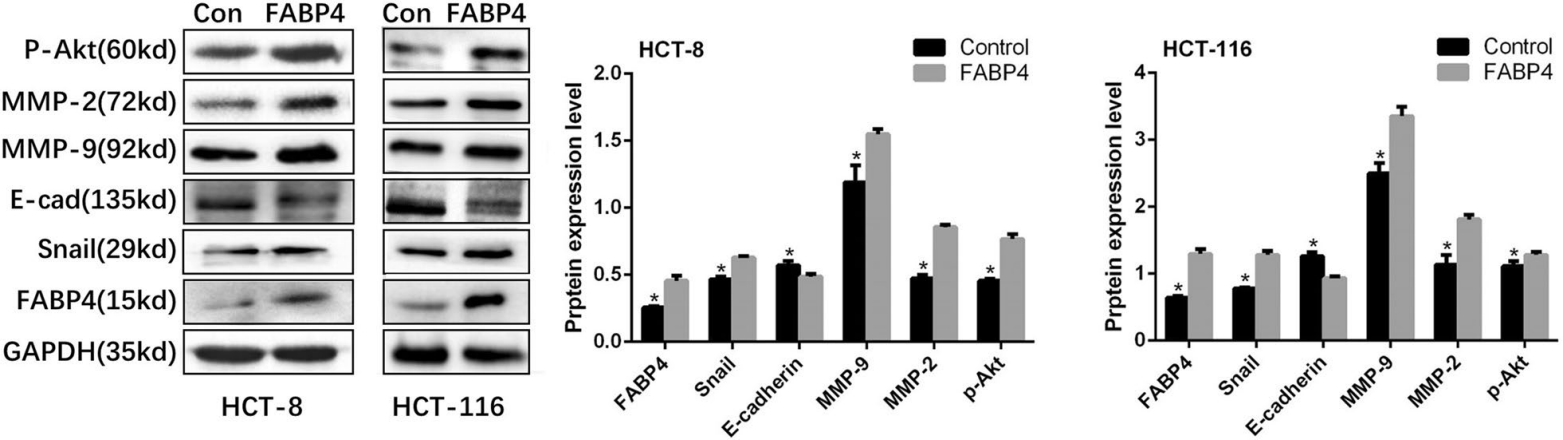
FABP4 enhanced EMT in colon cancer cells through AKT pathway. Western blot was performed to detect the protein expression of Snail, E-cadherin, MMP-2, MMP-9 and p-Akt, and the band densitometry analysis was carried out. All the samples were prepared in triplicate, and all experiments were repeated for at least three times. *P < 0.05

EMT has been confirmed as a key mechanism underlying tumor metastasis [9]. To explore the relationship between FABP4 and EMT, the expressions of EMT-related protein markers, such as Snail, E-cadherin, MMP-2 and MMP-9, were determined by Western blot test. The gray values of Snail, MMP-9 and MMP-2 in FABP4-overexpressed HCT-8 cells were 1.35 ± 0.01, 1.30 ± 0.08, 1.81 ± 0.02 times of those in control group, respectively, and the gray value of E-cadherin in FABP4 overexpression group was about 85% of that in control group (Fig. 6, *P* < 0.05). The EMT was significantly enhanced in FABP4-overexpressed colon cancer cells, as evidenced by the upregulation of Snail, MMP-2 and MMP-9, and the downregulation of E-cadherin. Similar results were obtained from HCT-116 cells. To evaluate the effect of FABP4 on AKT, the expression of p-Akt was detected by Western blot test. The gray value of p-Akt in FABP4-overexpressed HCT-8 cells was 1.15 ± 0.05 times of that in the control group (Fig. 6, *P* < 0.05). Similar results were seen in HCT-116 cells. Overall, these results indicated that FABP4 enhanced the EMT in colon cancer cells, probably through AKT pathway.

## Discussion

The tumor microenvironment takes on dynamic profiles as the tumor develops [18–20]. Tumor patients may also display metabolic disorders, including the abnormal adipokine secretion in the tumor tissue, which turn tumor microenvironment adaptable for tumor invasion and metastasis [5, 20]. Studies have shown that cancer-associated adipocytes can regulate the progression of tumors, such as hepatocarcinoma, ovarian, and breast cancers, at least partially by affecting energy metabolism [3, 5, 21–23]. For ovarian cancer, adipocyte co-culture directly led to transfer of lipid from adipocytes to ovarian cancer cells and promoted tumor growth, suggesting that adipocytes are important component in the tumor microenvironment [3, 5, 7].

In our study, we observed lipids accumulated around the colon cancer cells, as shown by the higher triglyceride concentration in colon cancer tissues. Lipids mainly include triglycerides, glyceryl phosphatide, cholesterol and so on. Triglyceride makes up the majority of lipids, and can be decomposed into fatty acids which is important energy supply material [24, 25]. Studies have shown that lipids are mainly synthesized with fatty acids in the tumor, stored in lipid droplets in cytoplasm, and utilized by the tumor to support its own growth [26]. Our study found that co-culture with adipose tissue extract significantly enhanced the intracellular lipid accumulation in colon cancer cells, suggesting the fatty acid transport and lipid synthesis were active, which was consistent with the results of Nieman et al. [3]. Fatty acid transport between tumor cells and adipocytes and its metabolism are complex systems [3, 19], in which FABP4 plays a vital role. It has been demonstrated that FABP4 was up-regulated in most tumors, such as prostate cancer and breast cancer [27, 28]. In our study, we found that the transcription level of FABP4 in colon cancer tissues was significantly increased, indicating the enhanced lipogenesis ability of colon cancer. Immunofluorescence verified that the FABP4 protein could be taken up by colon cancer cells, and its overexpression significantly enhanced the lipid accumulation. The results indicated that FABP4 promoted lipid transport.

Our study showed the migration and invasion of colon cancer cells were significantly enhanced when co-cultured with extract of adipose tissues or overexpressed FABP4, but the proliferation did not change. BMS309403 weakened the migration and invasion of colon cancer cells. Uehara et al*.* [16] found FABP4 inhibitor reduced the subcutaneous growth and lung metastasis of prostate cancer cells in mice. In our vivo experiments, the mice injected with FABP4-overexpressed HCT-116 cells developed more and severer lung metastases. The results above confirmed that FABP4 could promote colon cancer metastasis in vivo and in vitro.

Glycerol and free fatty acids are reliable indicators of lipid metabolism [29]. Our study found that the concentrations of glycerol and free fatty acids were increased significantly in colon cancer cells co-cultured with extract of adipose tissues, which indicated the lipid metabolism had been enhanced. The consumption of ATP and LDH was enhanced significantly in colon cancer cells co-cultured with extract of adipose tissues, revealing that the energy metabolism had been accelerated. At the same time, when the FABP4 protein was inhibited by specific inhibitor BMS309403, the lipid metabolism and energy metabolism were suppressed significantly. Therefore, we came to the conclusion that FABP4 can promote lipid metabolism and energy metabolism. GO and KEGG are bioinformatics databases used for computational prediction of highly complex cellular processes and organism behavior, including signaling pathways and gene function. In our experiment, bioinformatics analysis discovered that FABP4 was enriched in 11 pathways related to metabolism, including lipid metabolism and energy metabolism, which further proved the important role of FABP4 in energy metabolism of colon cancer.

Adipocytes are also involved in the EMT of tumor cells, a key mechanism driving tumor metastasis [8, 9]. During EMT, epithelial cells acquire mesenchymal properties in structure and function, which facilitates the tumor cells to spread. EMT-inducing transcription factors, such as Snail, regulate the expressions of E-cadherin and genes associated with mesenchymal phenotypes (including MMPs), thus leading to the formation of migratory structure and the degradation of the extracellular matrix [17, 30]. In our study, the EMT phenotype markers changed significantly in FABP4-overexpressed colon cancer cells: the upregulation of Snail, MMP-2 and MMP-9, as well as the downregulation of E-cadherin. Therefore, FABP4 could endow colon cancer cells with mesenchymal properties, characterized by reducing cell-to-cell adhesion regulated by E-cadherin, and increasing the expressions of mesenchymal biomarkers. In summary, FABP4 could promote colon cancer metastasis by inducing EMT.

Recent studies have emphasized the role of the AKT signaling pathway in the induction of EMT in different cancer cells [31]. AKT, a Serine/Threonine kinase, is a key component in numerous processes, can phosphorylate and then regulate vital downstream effector molecules, including FOXO, mTOR, GSK3b, and promote cancer cell growth, metabolism and survival and induce EMT and metastasis [32]. Phosphorylation of AKT is a key step in the activation of AKT pathway. Studies have shown that hyperactivation of AKT can lead to EMT in in vitro and in vivo CRC models [33]. In our study, we found the level of p-Akt was up-regulated in colon cancer cells with FABP4 overexpression. According to these results, we speculated that FABP4 may enhance the metabolism and EMT in colon cancer cells partly by activating AKT pathway.

## Conclusion

**Fig.7:**
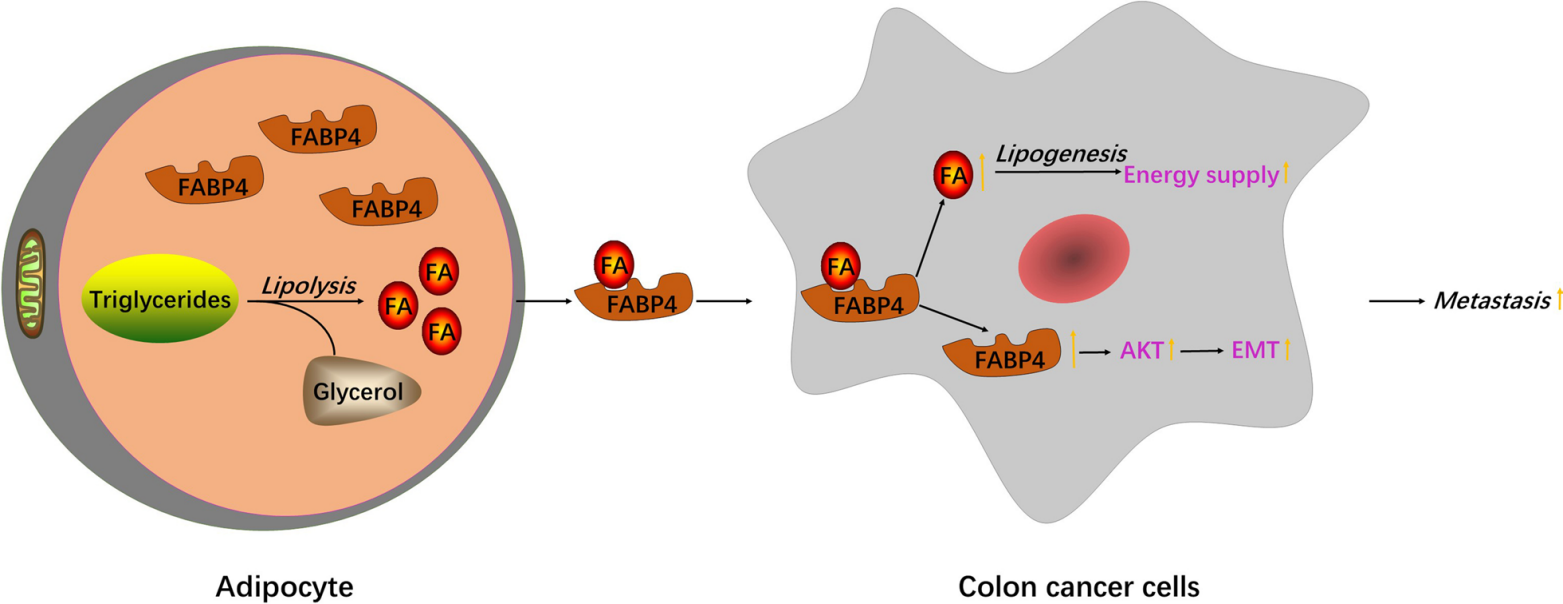
FABP4 promoted FAs transport to colon cancer cells, provided energy for tumor cells, and promoted the migration and invasion of colon cancer cells by activating EMT and AKT

FABP4 overexpression could increase FAs transport to enhance energy and lipid metabolism, and activate AKT pathway and EMT to promote the migration and invasion of colon cancer cells (Fig. 7). Our findings offer a new potential therapeutic target against colon cancer progression. However, how FABP4 regulates EMT downstream signaling cascades in colon cancer cells needs to be resolved with further experiments.

## Supplementary information

**Additional file 1: Figure S1.** semi-quantitative evaluation of immunocytochemistry and immunohistochemistry, and the verification of FABP4 overexpression at mRNA and protein levels.

## Data Availability

Please contact corresponding author for data requests.

## Abbreviations

FABP: Fatty acid binding protein
EMT: Epithelial–mesenchymal transition
FA: Fatty acid
NEFA: Nonesterified fatty acid
qRT-PCR: Real time-polymerase chain reaction
EGFP: Enhanced green fluorescent protein
MMPs: Matrix metalloproteinase
