# Supplementary material for: FABP4 promotes invasion and metastasis of colon cancer by regulating fatty acid transport
Source: Cancer Cell Int. 2020 Oct 19;20:512. doi: 10.1186/s12935-020-01582-4 (PMC7574203; doi:10.1186/s12935-020-01582-4)
Supplement: Supplementary file 1 — Additional file 1: Figure S1. semi-quantitative evaluation of immunocytochemistry and immunohistochemistry, and the verification of FABP4 overexpression at mRNA and protein levels. [file 12935_2020_1582_MOESM1_ESM.docx]

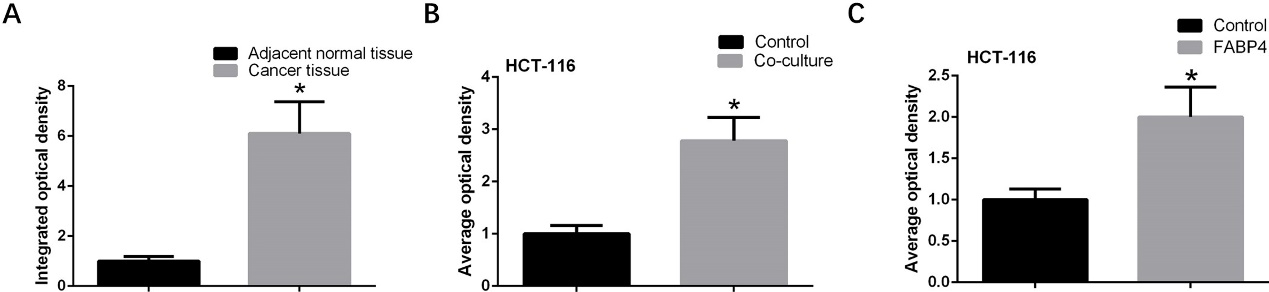


Fig. S1 Semi-quantitative evaluation with immunocytochemistry and immunohistochemistry. (A) Relative optical density of lipids in human colon cancer tissues and adjacent normal tissues. (B, C) The average optical density of lipids in HCT-116 cells. All the samples were prepared in triplicate, and all experiments were repeated for at least three times. **P* < 0.05


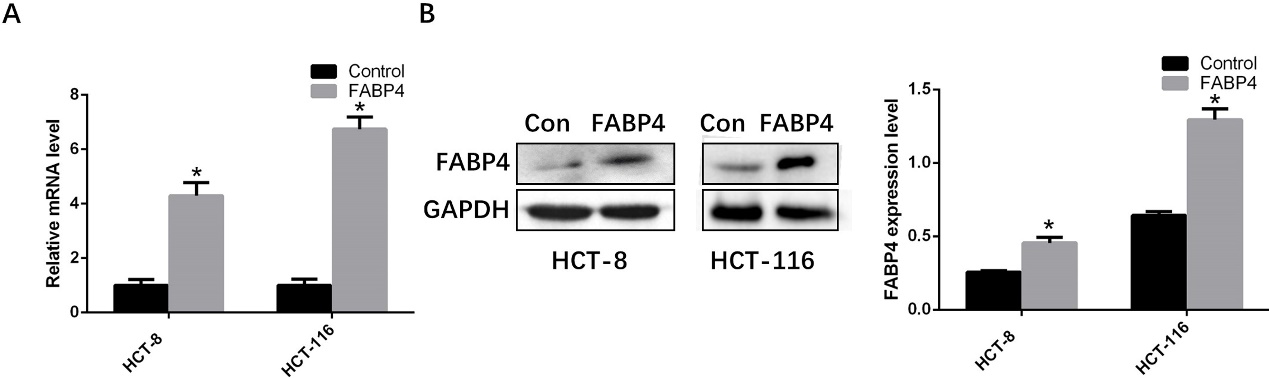


Fig. S2 The verification of FABP4 overexpression at mRNA and protein levels. (A) The mRNA level of FABP4 in colon cancer cells (FABP4-overexpressed group and control group). (B) Western blot analysis was performed to detect the protein expression of FABP4 in colon cancer cells (FABP4-overexpressed group and control group), and the band densitometry analysis was carried out. All the samples were prepared in triplicate, and all experiments were repeated for at least three times. **P* < 0.05
